# *In vitro* Elution of Penicillin, Ampicillin, Tetracycline, Tulathromycin, and Florfenicol From Plaster of Paris Beads

**DOI:** 10.3389/fvets.2020.585423

**Published:** 2020-11-30

**Authors:** Paul Merkatoris, Jennifer Schleining, Adam Krull, David Borts, Virginia Fajt

**Affiliations:** ^1^Department of Veterinary Diagnostic and Production Animal Medicine, College of Veterinary Medicine, Iowa State University, Ames, IA, United States; ^2^Department of Large Animal Clinical Sciences, College of Veterinary Medicine & Biomedical Sciences, Texas A&M University, College Station, TX, United States; ^3^Department of Veterinary Physiology and Pharmacology, College of Veterinary Medicine & Biomedical Sciences, Texas A&M University, College Station, TX, United States

**Keywords:** Plaster of Paris, antibiotics, wound infection, elution, cattle

## Abstract

The objectives of this study were to report a recipe for making antibiotic impregnated Plaster of Paris (AI-PoP) beads using penicillin, ampicillin, tetracycline, tulathromycin, and florfenicol and to determine the *in vitro* elution rates of those antibiotics in the beads. The AI-PoP beads were made using Plaster of Paris powder, antibiotic, and water, cured for 24 h, sterilized by ethylene oxide, and stored up to 5 months before testing. For each antibiotic, 20 beads were combined with bovine serum in sterile tubes and incubated at 37°C on a rocker. Serum was replaced at intervals over the 14 days study period, and antibiotic concentrations were determined by high pressure liquid chromatography with mass spectrometry. Separately, in a proof-of-concept study, the growth of *E. coli* and *T. pyogenes* in eluent from 10 beads for each antibiotic was quantified by flow cytometry. Antibiotic was detected in AI-PoP bead eluent for 14 days for all but the ampicillin beads, for which antibiotic was detected for 8 days. The concentration of antibiotic in eluent was greater than the minimum inhibitory concentration (MIC) of tested bacteria for the entire study period for penicillin, tetracycline, tulathromycin, and florfenicol. The concentration of ampicillin remained greater than the MIC of *E. coli* for 4 days and *T. pyogenes* for 6 days. The colony forming units (CFU)/ml of live *E. coli* and *T. pyogenes* was reduced over a 72-h period by 1–3 log_10_ CFU, with the exception of tetracycline, which reduced CFU/ml of *T. pyogenes* by <log_10_ CFU. AI-PoP beads containing penicillin, tetracycline, tulathromycin, and florfenicol elute antibiotic well-above the MIC of selected isolates for the 2 weeks study period. These antibiotics show promise for delivery in joint and wound infections.

## Introduction

Synovial sepsis, post-operative surgical site infections, and complicated wounds are a devastating cause of increased morbidity and mortality in large animal veterinary patients and can be career or even life-ending (1–3). While systemic antibiotics are the mainstay of treatment, complementary local antimicrobial therapies are often employed. These adjunct therapies are especially useful in the treatment of osteomyelitis, synovial sepsis, and surgical site infections (4–6). The benefits of local therapy include the ability to achieve local drug concentrations many times above bacterial minimum inhibitory concentration (MIC), avoidance of high systemic doses of antibiotics, and cost effectiveness (7–9).

The options for local antimicrobial therapy include intravenous and intraosseous regional limb perfusion (10, 11) and sustained release of antimicrobials eluted from implanted carriers such as collagen sponges (12), polymethylmethacrylate (13, 14), plaster of Paris (PoP) (7–9), constant rate infusion systems (15), and hydroxyapatite cement (13). Regional perfusions can achieve antimicrobial concentrations many times higher than the MIC of common pathogens, however use is limited to the distal extremity due to the need to place a tourniquet proximal to the area of interest and because high concentrations of drug are generally not maintained for very long after tourniquet removal. Antibiotics reported for use in regional perfusions in cattle include florfenicol, ampicillin-sulbactam, marbofloxacin, and ceftiofur (16–19). Constant rate infusion systems are also able to achieve high antimicrobial concentrations, however breakage or blockage of the intrasynovial catheter is common (15). Non-biodegradable polymethylmethacrylate implants are easy to use, however removal may be necessary. In addition, creating the implant produces an exothermic reaction, thus only heat stable antibiotics may be used (15). Biodegradable implants, including collagen sponges, hydroxyapatite cement, and PoP, offer the advantage of not requiring removal and being potentially more biocompatible. Purified type I collagen sponges are biocompatible, biodegradable, and are characterized by low immunogenicity (12). Collagen sponges and hydroxyapatite cement have the disadvantage of being more expensive than PoP. Plaster of Paris, or calcium sulfate hemihydrate, is inexpensive, readily available, and easy to use for local antimicrobial delivery (15). In addition, it is biocompatible, biodegradable, and osteoconductive when used in fracture repair and established osteomyelitis, providing a scaffold on which new bone formation can occur (7, 15). The *in vitro* elution of antibiotic from PoP beads has been reported for gentamicin, amikacin, clindamycin, enrofloxacin, vancomycin, tobramycin, and cefazolin (7–9, 20, 21). These studies show that the elution profile is different among antibiotics.

The use of antibiotic impregnated PoP (AI-PoP) beads constitutes an extra-label drug use under the Animal Medicinal Drug Use Clarification Act of 1994, which also prohibits or limits the use of previously studied drugs in food producing species. Antibiotics elected for use in this study include penicillin, ampicillin, tetracycline, tulathromycin, and florfenicol. These antibiotics were chosen because they are commonly used in food animal practice and extra-label use may be allowed.

The objectives of this study were to (1) report a recipe for making AI-PoP beads using penicillin, ampicillin, tetracycline, tulathromycin, and florfenicol and (2) determine the *in vitro* elution properties of the AI-PoP beads over 14 days.

We hypothesized that (1) the elution of all antibiotics from the AI-PoP beads would have a rapid initial phase and complete elution within 2 weeks and (2) the initial concentrations of antibiotic in the eluent would exceed the MIC of bacterial isolates used.

## Materials and Methods

Based on methods previously described (7, 9), AI-PoP beads were made on an open benchtop as follows: 20 grams of non-sterile PoP powder (Plaster of Paris, DAP Products Inc., Baltimore, MD) were thoroughly mixed with antibiotic powder or liquid in a specimen cup. When needed, water was added to achieve a pourable mixture (see Table 1 for weights and volumes used). Ampicillin (Polyflex®, Boehringer Ingelheim Vetmedica Inc., St. Joseph, MO) and tetracycline (TC Vet 324™, Vetone, Manufactured for: MWI, Boise, ID) powder were used, whereas penicillin G procaine (PenOne Pro™, Norbrook Laboratories United, Newry, Northern Ireland), tulathromycin (Draxxin®, Zoetic Inc., Kalamazoo, MI), and florfenicol (Norfenicol®, Norbrook Laboratories Limited, Newry, North Ireland) were in solution. All AI-PoP bead ingredients were chosen for being inexpensive and easily accessible to general practice veterinarians, as it was desired to create a bead recipe that could be replicated and used in a general practice setting. Bead recipes were determined by maximizing the amount of antibiotic used with the outcome of an appropriately hardened bead, as too much antibiotic resulted in a bead that did not harden appropriately. In this study, all PoP powder used was derived from the same carton, all water was obtained from the same source in the hospital, and all antibiotics were derived from the same bottle or vial. All bead types used experimentally were created in a single batch, thus eliminating the need to control for variability in lot numbers. Each mixture was poured into a silicone candy bead mold (Chicago Culinary FX, Westchester, IL) and a wooden tongue depressor was used to completely fill the wells. The use of a silicone bead mold allowed standardization of bead size in this study. The mold created uniform, 6 mm diameter beads, yielding a surface area of 113 mm^2^ and a volume of 113 mm^3^. The AI-PoP beads were allowed to cure in the mold for 24 h at room temperature (with the exception of penicillin beads, which were cured at 4°C), sterilized by ethylene oxide, and stored at room temperature (with the exception of penicillin beads, which were stored at 4°C based on storage conditions identified on the drug label). Testing was completed within 5 months of AI-PoP bead manufacturing.

**Table 1:** Antibiotic impregnated Plater of Paris bead recipes.

	**Penicillin**	**Tetracycline**	**Ampicillin**	**Tulathromycin**	**Florfenicol**
PoP powder (g)	20	20	20	20	20
Antibiotic	12 ml	2,000 mg	2,000 mg	3 ml	3 ml
Water (ml)	0	8	9	5	6
Total antibiotic	3.6 MIU	2,000 mg	2,000 mg	300 mg	900 mg
Yield (beads)	132	134	121	108	116
Bead concentration (per bead)	27 KIU	14.9 mg	16.5 mg	2.78 mg	7.76 mg
Total antibiotic tested (mg)	288 MIU	1,192 mg	1,320 mg	222.4 mg	620.8 mg

For each antibiotic, 20 AI-PoP beads and seven milliliters of bovine serum (Lampire Biological Laboratories, Inc., Pipersville, PA) were aseptically transferred to individual sterile polypropylene test tubes. The eluent from each AI-PoP bead type was tested in quadruplicate; four test tubes of AI-PoP beads were tested for each antibiotic. The number of beads, choice of serum, and volume of serum were chosen based on previous studies (7, 9) and because it was sufficient to immerse the beads in serum. The minimal volume necessary to submerge the 20 beads in the 50 mL polypropylene test tubes was 5 mL. Two additional mL of bovine serum were added during the first 4 days of the experiment to mimic the increase of effusion occurring in the early phase of the infection. The tubes were maintained in a rotating incubator at 37°C, except when removing eluent and adding serum. The entire volume of eluent was removed at specified time points and replaced with fresh bovine serum. A volume of 7 mL was initially used at 4, 8, 12, 24, 36, 48, and 72 h. This volume was subsequently reduced to 5 mL at 96, 120, 144, 192, 264, and 336 h in order to mimic the reduction of effusion that clinically occurs during the healing period. Eluent samples were stored at −70°C until analyzed (~1 month).

Concentrations of five antibiotics were determined using high-pressure liquid chromatography with mass spectrometry detection after protein precipitation of samples with acetonitrile. High-pressure liquid chromatography with mass spectrometry detection was performed using an Agilent 1100 Pump, column compartment, and autosampler (Santa Clara, CA, USA) coupled to an ion trap mass spectrometer (LTQ, Thermo Scientific, San Jose, CA, USA). Serum samples, serum spikes, serum quality controls, and bovine serum blanks, 50 or 100 μL, depending on calibration curve concentrations of the antibiotic, were mixed with 450 μL of acetonitrile to precipitate serum proteins. The acetonitrile contained an internal standard at a concentration of either 250 or 1,000 ng/mL, depending on calibration curve concentrations of the antibiotic. The samples were vortexed for 5 s and centrifuged for 10 min at 7,500 rpm to sediment the protein pellet. A portion, 20 or 50 μL, of the supernatant was then diluted with water to 1 mL of volume in an autosampler vial. Further dilution of the samples, if needed, was done with blank serum that had been precipitated and diluted identically as the samples. These dilutions were done in autosampler vials fitted with glass inserts and dilutions were 1:5 or 1:10. The vials were then centrifuged at 2,400 rpm prior to analysis. All the quality control samples passed the tolerance of being within ± 15% of the nominal concentration. The limit of quantitation of the analysis was 0.5 μg/mL with a limit of detection of 0.1 μg/mL.

The mean observed peak of antibiotic elution was calculated for each antibiotic. The amount of antibiotic eluted was displayed in two different ways: first, as the amount of antibiotic eluted in the first 72 h, expressed as a percent of antibiotic released over the 2-weeks study period, and second, as the total amount of antibiotic released over the 2-weeks study period, expressed as a percent of total incorporated antibiotic. The concentration of antibiotic within the eluent was compared to the MICs of the bacterial isolates used in this study. Some authors consider time dependent antibiotics, such as those used in this study, to be most efficacious when concentration is maintained above the MIC for at least 50% of the dosing interval (22), thus, we chose to compare the antibiotic concentration to the MIC of the selected isolates. The elution half-life was calculated based on the log-linear regression of the terminal portion of the concentration-time curve using standard pharmacokinetic software (Certera Phoenix 64 8.1.0.3530). The elution half-life is the interval of time required for the concentration of antibiotic to be decreased by 50%.

The quantification of bacteria subjected to AI-PoP eluent was evaluated against two bacterial isolates, an American Type Culture Collection strain of *E. coli* and a clinical isolate of *T. pyogenes*. These are two of the most common isolates in cattle from cases of synovial sepsis (23). Together they encompass a broad spectrum of bacteria, *T. pyogenes* being a slow growing Gram-positive facultative anaerobe, and *E. coli* being a fastidious Gram-negative facultative anaerobe (24, 25). In this proof-of-concept phase of the study, a single test of each AI-PoP and bacteria combination was completed. Using the McFarland standard, colonies of pure cultures of *E. coli* and *T. pyogenes* were used to inoculate test tubes containing ten AI-PoP beads and five milliliters of Mueller Hinton (MH) broth to a concentration of 10^7^ CFU/mL. This concentration was elected because it was the most concentrated inoculum that could be reasonably achieved using these methods. It has been shown that the risk for surgical site infection is markedly increased when contaminated with more than 10^5^ microorganisms per gram of tissue (26). Thus, the inoculation dose of 10^7^ was considered sufficient to represent clinical infection. A single negative control test of MH broth lacking AI-PoP beads was also inoculated at the same concentration for each bacterium. Following inoculation, the tubes were maintained in a rotating incubator at 37°C. Twenty-four, 48, and 72 h after inoculation, a one milliliter sample of eluent was obtained for quantification of live and dead bacteria by flow cytometry, and the volume was replaced with sterile MH broth. Live and dead bacteria in samples were stained for quantification by flow cytometry using a commercially available kit (Molecular Probes, Inc.; Eugene, OR). Samples were prepared according to manufacturer's instruction, then immediately transported to the flow cytometry laboratory for quantification. The number of events in the bead region was set to 5,000 and the sample was assayed at 488 nm. The data were processed by framing the region around the live and dead populations to yield events data for live and dead bacteria. The bacterial culture density was calculated using outcome events data and dilution factors.

## Results

**Figure 1:**
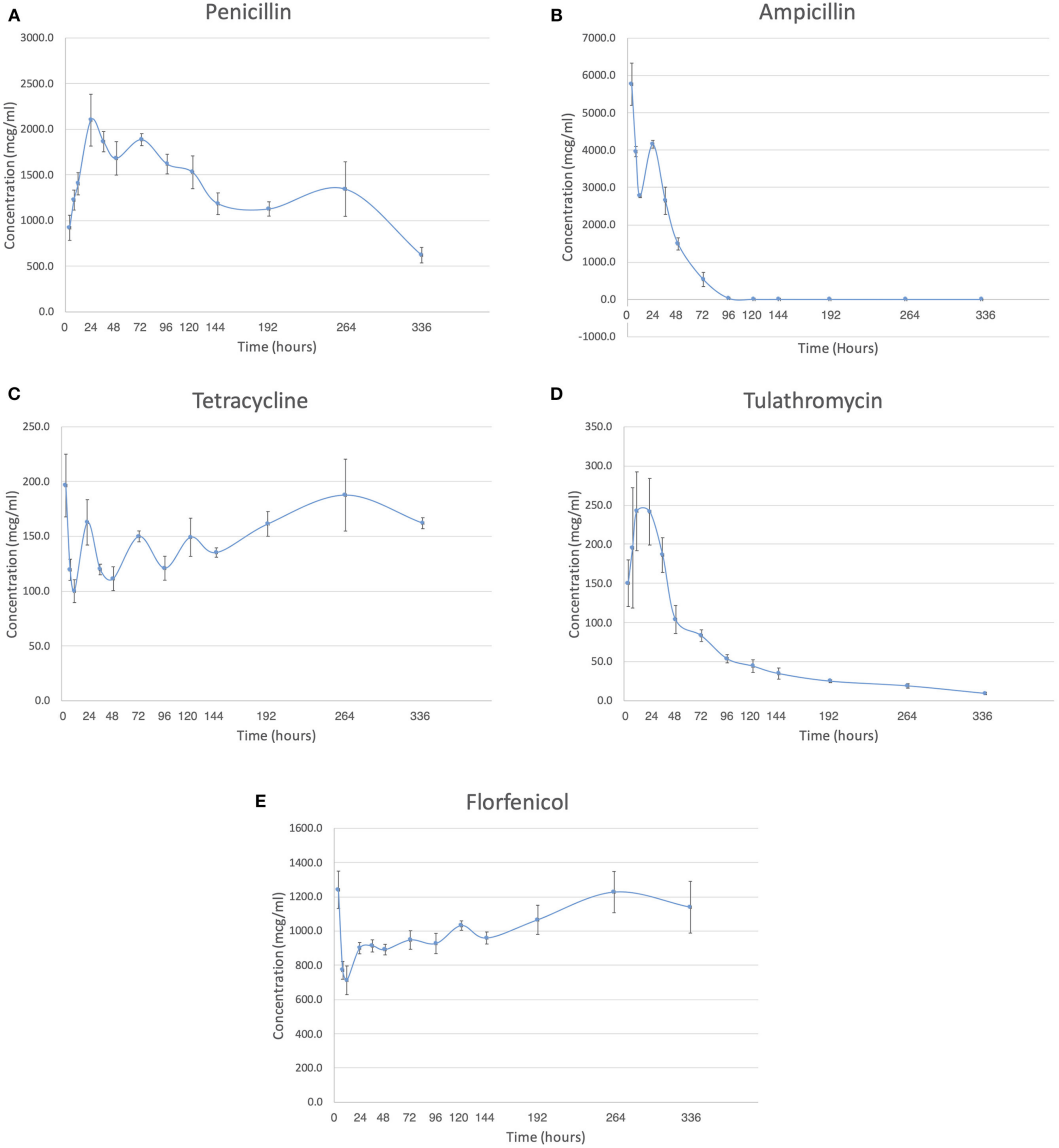
The concentration in mcg/ml is shown over the two-week study period for each antibiotic. **(A)** Penicillin. **(B)** Ampicillin. **(C)** Tetracycline. **(D)** Tulathromycin. **(E)** Florfenicol.

All AI-PoP beads eluted detectable concentrations of antibiotic for the 14-days sampling period, with the exception of ampicillin, which eluted detectable concentrations of antibiotic for 8 days. Mean observed peak ampicillin, florfenicol, and tetracycline concentrations occurred at 4 h, mean observed peak tulathromycin concentration occurred at 12 h, and mean observed peak penicillin concentration occurred at 24 h (Figure 1).

The amount of antibiotic released in the first 72 h, expressed as a percent of antibiotic released over the 2-weeks study period, was greatest for ampicillin and least for florfenicol (Table 1). The total amount of antibiotic released over the 2-weeks study period, expressed as a percent of total incorporated antibiotic, was greatest for florfenicol and least for tetracycline (Table 2).

**Table 2:** Mean percent elution in terms of total included antibiotic and antibiotic eluted over the 14 day study period.

	**14 d/total**	**Std dev**	**72 h/14 d**	**Std dev**
Ampicillin	45.2	2.1	99.9	0.04
Florfenicol	49.2	2.2	58.4	0.8
Penicillin	33.6	0.9	67.6	2.1
Tetracycline	3.8	0.1	59.4	1.4
Tulathromycin	16.8	1.8	89.9	1.5

The MIC data for each antibiotic and bacterial strain, as provided by the Iowa State University Veterinary Diagnostic Laboratory, is shown in Table 3. The concentration of antibiotic within the eluent reported at each time point was greater than the MIC of both *E. coli* and *T. pyogenes* for penicillin, tetracycline, tulathromycin, and florfenicol for the entire study period. The concentration of ampicillin remained greater than the MIC of *E. coli* for 4 days and *T. pyogenes* for 6 days.

**Table 3:** Minimum inhibitory concentration (micrograms/mL) of isolates used, provided by the Iowa State University Veterinary Diagnostic Lab.

	***E. coli***	***T. pyogenes***
Ampicillin	4.00	0.25
Florfenicol	4.00	1.00
Tetracycline	1.00	4.00
Penicillin	>8.00	<=0.12
Tulathromycin	8.00	<=1.00

Ampicillin had the shortest elution half-life, and penicillin had the longest (Table 4). The elution half-life of florfenicol and tulathromycin could not be calculated in any of the samples because the slope was not negative.

**Table 4:** Elution half-life (in hours) of antibiotics in AI-PoP beads incubated for 14 days in bovine serum.

	**Rep 1**	**Rep 2**	**Rep 3**	**Rep 4**	**Mean**
Ampicillin	8.8	9.4	12.0	8.5	9.6
Penicillin	248	236	185	216	221.2
Tulathromycin	101	101	124	95	105.4
Florfenicol	Could not be calculated because slope was not negative				
Tetracycline	Could not be calculated because slope was not negative				

**Figure 2:**
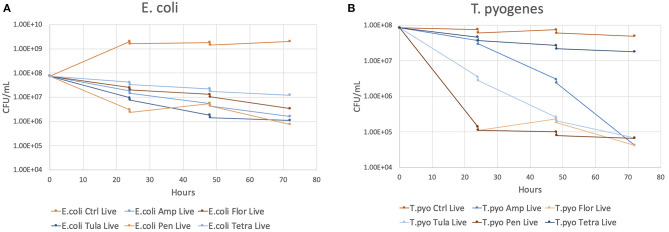
The CFU/ml of each antibiotic and the control at 24, 48, and 72 h of growth. **(A)** *E. coli*. **(B)** *T. pyogenes*.

The results of the flow cytometry proof-of-concept study are shown in Figures 2A,B. The decrease in concentrations observed at 24 and 48 h of growth represents the decrease in concentrations that resulted from adding 1 ml of MH broth to the culture tube following sampling. The colony forming units (CFU)/ml of *E. coli* is reduced by ~1–2 log_10_ CFU by 72 h for all antibiotics compared to the control sample. The CFU/ml of *T. pyogenes* is reduced by 3 log_10_ CFU for all antibiotics except tetracycline by 72 h compared to the control sample. CFU reduction in the tetracycline sample was <log_10_ CFU.

## Discussion

This study was successful in creating antibiotic impregnated beads using five different antibiotics. The first hypothesis, that the elution of all antibiotics from the AI-PoP beads would have a rapid initial phase and complete elution within 2 weeks, was rejected. Only the elution of ampicillin was complete within 2 weeks; the remaining antibiotics eluted antibiotic for the full 2-weeks study period. The second hypothesis, that the initial concentrations of antibiotic in the eluent would exceed the MIC of bacterial isolates used, was accepted. The concentration of antibiotic in the eluent exceeded the MIC of bacterial isolates for a minimum of 4 days.

The amount of antibiotic eluted by the AI-PoP beads was low in this study, ranging from 3.79 to 49.23%. The amount of antibiotic eluted has been shown to be proportional to dissolution of the bead, with the rate of dissolution being affected by increases in porosity (27). Porosity, determined by scanning electron microscopy (28), and rate of bead dissolution were not analyzed in this study because the ideal porosity and other biomaterials properties for AI-PoP beads remains unknown. The authors hypothesize that factors such as water solubility of the antibiotic and molecular binding properties with the PoP may have played a role in the differences in amount of antibiotic eluted. It has been speculated that factors such as storage temperature and humidity, length of time stored, and combination of different antibiotics used may have an effect on bead dissolution. The length of time stored prior to elution in this study was 5 months, and was 6 months in a previous study (7). It remains unknown what the maximum length of time these products may be considered shelf-stable; however, the products used in this study remained effective following 5 months of storage.

The elution pattern of penicillin, ampicillin, and tulathromycin in our study is similar to the previously reported elution pattern of most antibiotics (7, 9), characterized by an initial rapid release of antibiotic, subsequently followed by low concentration release for the remainder of the study period. The elution pattern of tetracycline and florfenicol is atypical in that a relatively constant level of elution was maintained for the entire study period. This pattern has previously been reported for enrofloxacin and was attributed to the drug having a low solubility in water (9). This may explain the elution pattern of florfenicol, as it also has a low solubility in water. However, tetracycline is soluble in water, and the authors suspect that there is an unknown molecular interaction between the PoP and tetracycline that is responsible for the elution pattern that was observed. One hypothesis is that oxytetracycline is known to chelate calcium ions, thus is may be chelating calcium present in the Plaster of Paris. The authors hypothesize that the longer duration of time between sampling times later in the study led to relatively higher levels of antibiotic in the eluent for tetracycline and florfenicol.

This study is consistent with previous reports that found a variable volume of liquid is needed for individual batches in order to obtain an appropriate antibiotic-PoP mixture consistency prior to filling the bead mold (7, 9). Because of individual properties associated with each antibiotic, each AI-PoP bead preparation was different. Because procaine penicillin G was used, penicillin beads were cured and stored in a refrigerator in accordance with the handling instructions of the drug. It is unknown whether this was necessary, or if curing and storage at room temperature would have been acceptable.

A sampling period of 2 weeks was chosen in this study. Based on the rapid elution of antibiotic from AI-PoP beads observed in a previous study using PoP beads (7), a similar elution profile was anticipated. However, we were surprised that in the current study, AI-PoP beads eluted antibiotic for the entire 14-days study period, with the exception of ampicillin. In previous studies using PoP beads, antibiotics such as gentamicin, amikacin, and clindamycin exhibited a rapid elution profile, while vancomycin, enrofloxacin, and tobramycin exhibited a prolonged release elution profile (7–9, 16).

Previous studies have used eluent to qualitatively describe bacterial inhibition (7, 8). Flow cytometry has been shown to be an accurate and rapid method of determination of the concentration of bacteria in a sample while distinguishing between live and dead organisms (29). This proof-of-concept portion of the study serves to show that flow cytometry can be used to quantitatively describe the effect of AI-PoP eluent on bacterial inhibition.

The choice of antibiotics for systemic administration should be based upon culture and antimicrobial susceptibility data, but because of a variety of factors including cost, time to achieve culture and sensitivity results, negative growth on cultures, and other reasons, the choice is often based upon clinical experience, empirical data, and commonly known bacterial isolates associated with specific conditions. The choice for local delivery of antibiotics is based upon similar criteria, although MIC breakpoints used to identify susceptible and resistant isolates are not predictive when antibiotic drugs are applied topically or locally, since achievable concentrations and effects on clinical outcome are unknown. Additional factors that could potentially influence the performance of local antimicrobials include bacterial burden, wound contamination, and wound exudate. It remains unknown how this *in vitro* work is to relate *in vivo*. There are many factors of *in vivo* infection that were not replicated by this study, including microbe-related risk factors such as biofilm, and host-related factors such as an immune response.

The antibiotics chosen for this study were selected because they are labeled for use in food producing species and their extralabel use may be allowed. Food producing species are subject to restrictions on drug use such as provisions within the Animal Medicinal Drug Use Clarification Act of 1994. It is recommended that the Food Animal Residue Avoidance Database be consulted for any extralabel drug use, including those described in this report. It is unknown what the serum or tissue concentrations of these drugs might be after application of AI-PoP bead, so caution should be used when predicting withdrawal times.

A limitation of this study is that elution was only investigated for 2 weeks. Elution data showed that a longer period would have been beneficial for penicillin, tetracycline, tulathromycin, and florfenicol. Another limitation is that the mechanical properties of the PoP beads were not characterized, and we were not able to provide further information regarding the rate of dissolution of the beads. A final limitation of this study is the absence of multiple specimens in the proof-of-concept study to determine the *in vitro* inhibition of bacteria by AI-PoP eluent.

Future studies should focus on the *in vivo* application of penicillin, tetracycline, tulathromycin, and florfenicol AI-PoP beads to corroborate the results of this *in vitro* study. These antibiotics appear to perform well in eluting antibiotic from AI-PoP beads and show promise for use in food animal species.

## Data Availability Statement

The raw data supporting the conclusions of this article will be made available by the authors, without undue reservation.

## Author Contributions

PM, JS, AK, and DB contributed to the design and completion of this study. VF performed the elution half-life analysis. All authors contributed to the writing, editing, and final approval of the manuscript.

## Conflict of Interest

The authors declare that the research was conducted in the absence of any commercial or financial relationships that could be construed as a potential conflict of interest.

## Abbreviations

AI-PoP: Antibiotic impregnated Plaster of Paris
MIC: Minimum inhibitory concentration
CFU: Colony forming units
PoP: Plaster of Paris
MH: Mueller Hinton.
